# The Effect of Metabolic Profile on Leptin, Adiponectin, and hs-CRP in Children and Adolescents with Type 1 Diabetes

**DOI:** 10.3390/children9081162

**Published:** 2022-08-03

**Authors:** Maria Kaza, Charalampos Tsentidis, Elpis Vlachopapadopoulou, Irine-Ikbale Sakou, Spyridon Karanasios, George Mastorakos, Kyriaki Karavanaki

**Affiliations:** 1 Diabetes and Metabolism Unit, 2nd Department of Pediatrics, National and Kapodistrian University of Athens, “P&A Kyriakou” Children’s Hospital, 115 27 Athens, Greece; isakuped@yahoo.gr (I.-I.S.); spyroskara97@yahoo.gr (S.K.); kkarav@yahoo.gr (K.K.); 2 Department of Endocrinology, Metabolism & Diabetes Mellitus, General Hospital of Nikaia-Piraeus “Agios Panteleimon”, 184 54 Piraeus, Greece; tsentidis@yahoo.gr; 3 Department of Endocrinology Growth and Development, “P&A Kyriakou” Children’s Hospital, 115 27 Athens, Greece; elpis.vl@gmail.com; 4 Endocrine Unit, National and Kapodistrian University of Athens, “Aretaieion” Hospital, 115 28 Athens, Greece; mastorakg@gmail.com

**Keywords:** adipokines, adolescence, pedometers, obesity, children

## Abstract

Adipokines are a superfamily of cell signaling proteins produced by the adipose tissue. This study’s purpose was to reveal the association of adipokines (leptin, adiponectin), hs-CRP, and IL-6 with well-known cardiovascular risk factors (lipid profile, diabetes control, obesity, physical activity) in children and adolescents with T1D. This cross-sectional study included 80 participants (36 boys) with T1D, aged (mean ± SD) 14.8 ± 3.4 years. Body Mass Index (BMI), metabolic profile, and level of physical activity were assessed (using pedometers) for evaluation of their effect on serum leptin, adiponectin, IL-6, and hs-CRP. Leptin levels were associated with BMI (beta = 0.184, *p* < 0.001), waist to hip ratio (beta = −2.017, *p* = 0.022), Low Density Lipoprotein-C (LDL-C) (beta = 0.021, *p* = 0.005), and fat mass (beta = 14.07, *p* < 0.001). Adiponectin was correlated with waist to height ratio (beta = 0.048, *p* = 0.006), ΒΜΙ (beta = −0.056, *p* = 0.005), and muscle mass (beta = −0.013, *p* = 0.020). Interestingly, hs-CRP was associated with weight (beta = 0.035, *p* < 0.001), ΒΜI (beta = 0.186, *p* < 0.001), fat mass (beta = 5.2859, *p* = 0.004), and muscle mass (beta = 0.027, *p* = 0.008). Multiple regression analysis of muscle mass unveiled associations with log hs-CRP (beta = −1.237, *p* = 0.014) and inverse IL−6 (beta = 18.57, *p* = 0.01). Finally, multiple regression models of fat mass unveiled associations with physical activity (7-day-total-step-count) (beta = −3.90 × 10^−7^, *p* = 0.027), Inverse IL-6 (beta = −0.1572, *p* = 0.009), and squared leptin (beta = 0.0077, *p* = 0.03). This study reports a positive association of leptin with LDL-C, BMI, fat mass, and hip circumference and a negative association of adiponectin with BMI and muscle mass. Finally, hs-CRP was associated with HbA1c, fat mass, and BMI. We propose that leptin, adiponectin, and hs-CRP could be used as prognostic indicators of cardiovascular risk in children with T1D.

## 1. Introduction

Type 1 diabetes (T1D) is a common chronic metabolic disease in children and adole- scents, characterized by destruction (autoimmune and idiopathic) of pancreatic endocrine β-cells, leading to complete insulin deficiency and consequently to an inability to maintain stable body blood glucose levels [1]. The Centers for Disease Control and Prevention (CDC) reported that between 2011–2012 children and adolescents (<20 years old) with new onset T1D in the US were approximately 17,900 [2]. According to 2021 estimations of the International Diabetes Federation (IDF), more than 1,200,000 individuals of 0–19 years old currently have T1D around the world [3], posing a major socioeconomic, morbidity, and mortality burden. Resent research data conclude that individuals with T1D are at higher risk of developing premature cardiovascular disease compared to the general population, and the risk of hospitalization is eight times higher for those diagnosed in childhood [4,5]. Although the correlation between diabetes and CVD is well established, the underlying causative mechanisms are not completely understood. The association of adipokines (leptin, adiponectin), hs-CRP, and IL-6 with well-known cardiovascular risk factors (lipid profile, diabetes regulation, obesity, physical activity) in children and adolescents with T1D is this study’s main purpose.

For the past few decades, there has been growing interest in investigating the role of adipokines in metabolism, a group of cell-signaling proteins (cytokines) produced mainly by the adipose tissue cells that hold a key role in the development of obesity. Leptin, a protein with a dual role as a hormone and as a cytokine, regulates bone metabolism and homeostasis through thermoregulation, while as a cytokine it promotes inflammatory response. Elevated leptin levels are found in the majority of obese patients, suggesting a state of leptin resistance [6,7]. Adiponectin on the other hand is an adipokine usually found in low levels in obese individuals, with anti-atherogenic, anti-diabetic, and anti-inflammatory properties [8].

IL-6 is a pro-inflammatory cytokine secreted by fibroblasts and endothelium, and to a large extent by adipose tissue cells. IL-6 holds a dominant role in the acute phase of inflammatory response, participates in the development of atherosclerosis, and constitutes a strong prognostic mortality marker along with hs-CRP and fibrinogen [9,10]. High sensitivity CRP, a nonspecific inflammatory marker found in dysfunctional vascular endothelium and early atheromatous lesions, is proven to be an independent prognostic marker of cardiovascular disease in the general adult population [9,11].

### Aim

This study’s main purpose was to reveal the association of adipokines (leptin, adiponectin), hs-CRP, and IL-6 with cardiovascular risk factors (lipid profile, diabetes control, obesity, physical activity) in children and adolescents with T1D.

## 2. Materials and Methods

### 2.1. Subjects

This is a cross-sectional cohort study assessing the metabolic profile (HbA1c, lipids, body composition), biochemical markers, and the level of physical activity of 80 children and adolescents (36 boys and 44 girls) with T1D, who attended the Diabetes and Metabolism Clinic of the 2nd Pediatric Department, National and Kapodistrian University of Athens, “P.& A.Kyriakou” Children’s Hospital, Athens, Greece during 2019 and 2020. This study took place in the above-mentioned Diabetes and Metabolism Clinic of the “P.& A.Kyriakou” Children’s Hospital, Athens, Greece. Inclusion criteria for participation in this study were: age 6–21 years and duration of T1D ≥ 1 year. The exclusion criteria were: children that did not attend the aforementioned Diabetes and Metabolism Clinic during 2019 and 2020, children with other types of Diabetes, children’s age younger than 6 years old or older than 21 years old, duration of T1D less than 1 year, children with mobile disabilities or other chronic untreated conditions (i.e., Addison Disease or lipodystrophies), children with acute febrile illness, and finally children on treatment with anticontraceptive pills, anabolic steroids, or statins.

### 2.2. Protocol

All participant data for the most recent laboratory examinations were derived from medical records (HbA1c, Thyroid-Stimulating-Hormone (TSH), biochemical and hematological markers) as well as their personal and family health history. Annual HbA1c was measured as the mean of three measurements within the past year. Participants’ blood pressure, pulse rate, height, and weight were measured in the clinic setting. BMI was calculated with Quetelet formula (BMI (kg/m^2^) = mass (kg)/height (m)^2^). Standard deviation score (SDS) BMI (z-score BMI) was evaluated using national normative data [12]. Pubertal status was assessed and categorized according to visual Marshall and Tanner scale by a single experienced examiner [13,14]. The percentage of fat and muscle mass was assessed with the Bioelectrical Impedance analysis (B.I.A, Tanita, Body Composition Analyzer, Type BC-418 MA). Physical activity of the participants was estimated for 1 week with the use of pedometers (Omron walking style IV). The result was documented as the number of total steps per week.

Serum blood samples were obtained after an overnight fast to determine levels of leptin, adiponectin, IL-6, and hs-CRP. Serum blood samples were centrifuged immediately after collection and stored in −70 °C until analysis.

The laboratory parameters of study subjects were documented from their recent visit at hospital if within 1 week from the time the pedometers were placed on participants and blood samples for adipokines, IL-6, and hs-CRP were obtained. If not, further blood tests were done within the next week.

### 2.3. Assays

After careful and slow thawing: (i) Serum leptin samples were processed with a leptin ELISA kit (Biovendor 62100, Brno, Czech Republic), with an intra Assay Variation CV% of 4.2%, an inter Assay Variation CV% of 6.7%, and a detection Limit of 0.2 ng/mL; (ii) Adiponectin serum samples were processed with an ELISA kit (Invitrogen, Campus Vienna Biocenter 2, 1030, Vienna, Austria), with an intra Assay Variation CV% of 4.2%, an inter Assay Variation CV% of 3.1%, and a detection Limit of 0.012 ng/mL; (iii) an hs-CRP ELISA kit (HyCult Biotech, Frontstraat 2A, 5405 PB Uden) was used for hs-CRP serum samples, with an intra Assay Variation CV% of 4.1%, an inter Assay Variation CV% of 6.3%, and a detection limit of 0.4 ng/mL; and (iv) an IL-6 ELISA kit (Origene Tech., 9620 Medical Center Drive, 200, Rockville, MD, USA) with an intra Assay Variation CV% of 6.2%, an inter Assay Variation CV% of 7.2%, and a detection Limit of 0.3 pg/mL were used for IL-6 levels.

### 2.4. Ethics

Participation in the study was voluntary. All participants, or their parents for those younger than 18 years old, gave written informed consent. The confidentiality of medical information of patients was maintained. Due to the nature of the protocol, which is essentially a study of observation and not of intervention, no ethical issues arise. At any stage of the study, they reserved the right to withdraw their consent and leave the study.

### 2.5. Statistical Analysis

Data handling and statistical analyses were performed using SPSS (version 23) and STATA SE for Windows v11.2, (Stata Corp, Texas, TX, USA, 2012). Data were expressed as actual numbers (percentage proportions) for categorical variables and mean ± Standard Deviation (SD), median, interquartile range for numerical variables. All numerical variables were analyzed both graphically and statistically using Shapiro–Wilk criterion. While most variables were found to adequately approximate a normal distribution, mathematical transformation was necessary in the four variables of interest. Leptin was square root transformed, adiponectin and CRP were log transformed, and IL-6 was inverted.

Univariate linear regression models were used to study associations of leptin, adiponectin, hs-CRP, and IL-6 with demographic and with clinical and biochemical variables. Multiple linear regression models using the forward stepwise method were used to study the independent predictors of the aforementioned four variables. A *p*-value of < 0.05 was considered statistically significant.

## 3. Results

The demographic, clinical, and biochemical characteristics are described in Table 1 and Table 2, expressed as mean ± SD and median interquartile range. The mean ± SD age of T1D patients was 14.8 ± 3.4 years, the disease duration was 5.8 ± 4 years and HbA1c was 8.0 ± 1.41%. The study population, consisted of 45% males and 55% females, 13.7% of subjects were prepubertal and 86.3% were pubertal.

In univariate models, leptin was positively associated with gender, hip circumference (cm), BMI (kg/m^2^), z score BMI, fat-mass percentage, neutrophils percentage and LDL-C (mg/dL) and negatively associated with waist to hip ratio, leukocyte percentage, ferritin (ng/mL), and urea levels (mg/dL) (Table 3). Adiponectin was negatively associated with age, height (cm), weight (kg), waist circumference (cm), ΒΜΙ (kg/m^2^), muscle mass (kg), ferritin (ng/mL) and positively with waist to height ratio (Table 4).

Children’s most recent HbA1c (%) value was positively associated with BMI (kg/m^2^), (beta = 0.1236, *p* = 0.007) and z-score BMI (beta = 0.3759, *p* = 0.037), whilst average annual HbA1c (mean of HbA1c% measured in the last year) was associated with muscle mass (kg) (beta = 0.03033, *p* = 0.016), z-score BMI (beta = 0.348, *p* = 0.04), and BMI (kg/m^2^), (beta = 0.1252, *p* = 0.003). Fat mass (%) was associated with subjects’ age, (beta = −0.0054, *p* = 0.025), hip circumference (cm), (beta = 0.00337, *p* < 0.001), BMI (kg/m^2^), (beta = 0.00782, *p* < 0.001), z-score BMI (beta = 0.04023, *p* < 0.001), and waist to hip ratio (beta =−0.1339, *p* = 0.001). Muscle mass (kg) was associated with subject’s age (beta = 3.0184, *p* < 0.001), Tanner stage, (beta = 7.963, *p* < 0.001), height (cm), (beta = 0.34801, *p* < 0.001), weight (kg), (beta = 0.7724, *p* < 0.001), waist circumference (cm), (beta = 0.91185, *p* < 0.001), hip circumference (cm), (beta = 0.61683, *p* < 0.001), BMI (kg/m^2^), (beta = 2.3081, *p* < 0.001), and BMI z-score (beta = 3.1497, *p* = 0.043).

High sensitivity CRP was positively associated with HbA1c, mean annual HbA1c, age, Tanner stage, height (cm), weight (kg), waist circumference (cm), ΒΜΙ (kg/m^2^), z score BMI, fat mass percentage and muscle mass (kg), disease duration, neutrophils percentage and negatively with daily glucose measurements (Table 5). No association was found between IL-6 and other variables.

Multiple regression analysis models of muscle mass (kg), after applying the forward stepwise method for variable insertion and evaluation with demographic and anthropometric data unveiled associations with log hs-CRP (beta = −1.237, *p* = 0.014) and Inverse IL-6 (beta = 18.57, *p* = 0.01). The independent predictors of muscle mass were gender, BMI z score, age, Tanner stage, and waist circumference (cm) (Table 6).

Finally, multiple regression models of fat mass (%) unveiled associations with physical activity (assessed as total seven-day steps count), Inverse IL-6, and squared leptin levels. The independent predictors of fat mass were gender and z- score BMI, (Table 7).

Overall, multiple regression analysis revealed that in this study gender, body weight (kg), and BMI (kg/m^2^) were independent predictors of leptin. Age and waist to height ratio were independent predictors of adiponectin, while age and z score BMI were the respective independent predictors of hs-CRP (Table 8).

Interestingly, in multiple regression models after controlling for the effect of age, gender, weight, and BMI (kg/m^2^), a positive association of leptin with adiponectin was found [F (5,72) = 16.66, R2 = 0.53, overall *p* < 0.001, beta (95% C.I.) = 0.46 (0.02, 0.90), *p* = 0.038].

## 4. Discussion

The increasing prevalence of obesity around the world over the past few decades has shifted the interest of the scientific community toward adipose tissue as an endocrine organ. The aim of this study was to investigate the association of adipokines with physical activity, diabetes control, obesity, and inflammatory response in children and adolescents with T1D. Studies regarding adipokines and physical activity in the T1D pediatric population are scarce [15,16]. To the best of our knowledge, there is no study objectively assessing the level of physical activity with pedometers, metabolic profile, body composition, inflammatory and biochemical markers on serum leptin, adiponectin, hs-CRP, and IL-6 in children and adolescents with T1D.

This study highlights a strong positive association of leptin with BMI and fat mass in children and adolescents with T1D. Obesity has been correlated with higher levels of leptin, supporting the hypothesis that this hormone is mainly produced by white adipose-tissue cells [6,7,17,18]. Moreover, a recent study by Tsiroukidou et al. [7] revealed strong associations between leptin and cardiorespiratory fitness in overweight and obese pediatric populations, underlining the need for early metabolic assessment in order to protect against future cardiovascular complications.

Interestingly, this study found that leptin levels were higher in children with T1D with an increased hip circumference and lower in T1D children with a higher waist to hip ratio, indicating that leptin levels might be more dependent on non-abdominal adipose tissue. This finding is in accordance with Harke et al. study on adult males with T2D, where leptin levels were negatively associated with waist to hip ratio [19].

A sedentary life style and reduced physical activity have been correlated with higher levels of leptin in the past [20]. This study found no association between physical activity (assessed with pedometers) and leptin in children and adolescents with T1D. A positive correlation between serum leptin levels and LDL-C was revealed but not with the other parameters of lipid profile (triglycerides, lipoprotein-a (Lp(a)), total cholesterol) high-density-lipoprotein-c (HDL-C), consistent with the Wu D et al. study [21].

Leptin values were higher in females compared to males in the present study and, moreover, were positively associated with fat mass. Circulating estrogen levels and/or a higher BMI and fat mass in female participants could explain the above finding, in accordance with previous studies in healthy adults and T1D children and adolescents, respectively [22,23].

The results of the present study are consistent with a strong inverse relationship between leptin levels and serum leukocytes, ferritin, and urea. Although as a cytokine leptin is expected to promote the inflammatory response, a negative association between cellular immunity and leptin levels has also been described by La Cava et al. [24]. The significance of this finding requires further larger scale follow-up studies in order to be verified.

In the present study adiponectin was negatively associated with ferritin levels, with no association with leucocyte count. Leptin and adiponectin are known to serve opposing roles involving the inflammatory response, which could explain the absence of the association in our study with leucocyte and neutrophil counts, observed for leptin. An inverse relationship between leptin and adiponectin has been reported in adults with T2D and in patients with obesity and coronary artery disease [17,19]. Leptin is a pro-inflammatory cytokine responsible for the upregulation of factors involved in the inflammatory pathway of T2D (i.e., TNF-α, IL-6) [6], while anti-inflammatory properties are attributed to adiponectin [6].

Interestingly, this study unveiled a positive association of leptin with adiponectin in multiple regression models in children and adolescents with T1D. Previous studies in T1D children [25], reported lower leptin and higher adiponectin levels in T1D newly diagnosed adolescents, which have changed overtime possibly as a result of lifestyle modifications (diet, exercise).

Adiponectin is an adipokine known for its anti-atherogenic, anti-diabetic, and anti-inflammatory properties [17]. A positive association of adiponectin with waist to height ratio and an inverse relationship with weight, waist circumference, and muscle mass was revealed in T1D children and adolescents in the present study. In agreement, previous studies describe an inverse relationship of adiponectin to obesity and hyperinsulinemia in pediatric populations [8,26].

In contrast to previous studies with T1D children [27], in the present study there was no association between adiponectin and physical activity or glycemic control, possibly because these associations refer to the total study population and not only to the subgroup with regular physical activity. In addition to that, a particularly interesting negative association is outlined in this study between adiponectin, diabetes duration, and ferritin levels. This association could be attributed to the inflammatory process triggered by T1D in relation to the anti-inflammatory properties of adiponectin [17,28].

Although females have been reported to have higher adiponectin levels [17], in the present study no association between adiponectin and gender was found. Moreover, a negative correlation of adiponectin with age and height was found in disagreement with previous studies, a finding that could be associated with a chronic inflammatory state due to the prolonged disease duration of T1D in older children [29].

IL-6 contributes to the development of atherosclerosis, inflammation, and cardiovascular disease [9]. It is noteworthy that in this study IL-6 was negatively associated with muscle mass. This finding shows the beneficial effect of physical activity on muscle mass and IL-6 levels during adolescence, eventually reducing cardiovascular risk. Moreover, lower levels of IL-6 have been associated in literature with increased physical activity in adults [11,30,31].

Interestingly, we found a strong positive association with hs-CRP and neutrophils, possibly attributed to the chronic inflammatory process associated with T1D [32]. Increased value of hs-CRP is a strong indicator of endothelial dysfunction, evident in even the non-diabetic population, with increased cardiovascular risk or with chronic kidney disease [33,34]. In the present study hs-CRP was strongly positive related to obesity mar-kers (increased weight, abdominal circumference, hip circumference, ΒΜΙ, and fat mass) and negatively associated with muscle mass. In agreement with the current results, the Pérez-Segura et al. study on children with T1D found that hs-CRP levels were correlated to BMI and waist circumference [35]. Furthermore, hs-CRP was elevated in T1D children compared with controls of the same BMI [35]. These results indicate that even at such a young age, increased body weight has an additional impact on the endothelial function and it is already affected by the presence of diabetes, highlighting the importance of weight surveillance in the aforementioned population [18,36].

In previous studies on the general population, increased physical activity was associated with a lower CRP level (19–35%) than those with a sedentary life style [9,11]. Instead, in our study on T1D children and adolescents, physical activity was not related to hs-CRP, most likely due to the chronic inflammatory state caused by diabetes.

Puberty is a critical period of development, during which secondary sexual cha-racteristics and reproductive capability are acquired. Amid this unique period, several hormonal changes take place in the human body, such as the development of the hypothalamic–pituitary–adrenal (HPA) axis [37]. A positive association was revealed in the present study of hs-CRP with age, progressed Tanner stage, and height. The aforementioned results could be explained by the previously described positive association of hs-CRP with disease duration and/or by the inflammatory process, as described by Stumper et al. du-ring the time of adolescence [38]. This interesting finding points out a possible and extremely important correlation between puberty/adolescence and the inflammatory process.

In the present study, most recent HbA1c and mean annual HbA1c were both positively associated with BMI and a BMI z-score. These findings are in agreement with studies in adults with T1D, associating metabolic syndrome with poor glycemic control [39]. The study of Hovestadt et al. on T1D children and adolescents found BMI to be associated with higher HbA1c levels [40]. Furthermore, although physical exercise and improved skeletal muscle mass have been known to increase insulin sensitivity and improve glycemic control in adults with T1D [41], the present study found an average annual HbA1c to be positively associated with muscle mass. As mentioned above, HbA1c levels have been associated with higher BMI levels in this study. Subsequently, increased BMI seems to be the confounding factor between HbA1c, muscle mass, and insulin sensitivity [42].

As anticipated, in the present study positive associations were found between fat mass and z-score BMI, hip circumference, and a consequent negative association with waist to hip ratio. Interestingly, fat mass was also negatively associated with age and positively associated with female gender. In opposition to the study in children and adolescents with T1D from Maffeis et al., this finding may be attributed to hormone derived changes in the body composition of both males and females as they enter puberty [43].

This study found a strong association between fat mass and inverted IL-6. This finding is consistent with recent literature performed in healthy adults, explaining the role of IL-6 as a chronic and a systemic inflammation marker, normally expected to increase in obesity, as the latter is well known to be a chronic inflammatory condition [44].

Another interesting finding of the present study was a strong and negative association of fat mass with the degree of physical activity assessed by pedometers (as total seven-day steps count), underlining the importance of exercise in children and adolescents with T1D in the prevention of obesity and obesity related comorbidities, such as well-known cardiometabolic complications [45].

The present study reports an association between muscle mass and age, Tanner stage, waist circumference, and z-score BMI. The above results are in agreement with the Bolaños et al. study on healthy children and adolescents [46], which are possibly attributed to the normal process of growth. In this study, muscle mass was found to be negatively associated with gender as physiologically males tend to have higher levels of skeletal mass than females, especially during puberty and adulthood [47].

Novelties of this study include the use of pedometers as an objective estimator of exercise; the use of the B.I.A scale to measure fat and muscle mass; the investigation of multiple associations between adipokines and the metabolic, physical, and biochemical profile of children and adolescents with T1D; the number of participants as well as the wide range of ages and body composition and Tanner stages. On the other hand, the main limitation of this study is the absence of control group.

The main findings may be summarized as follows: (i) Leptin was positively associated with adiponectin, LDL-C, BMI, fat mass, and hip circumference, (ii) Adiponectin was negatively related to BMI, weight and waist circumference, and muscle mass, and (iii) hs-CRP was strongly positively related to HbA1c, mean annual HbA1c, weight, ΒΜΙ/z score BMI, fat, and muscle-mass.

## 5. Conclusions

This study in children and adolescents with T1D unveils a positive association of leptin with well-known cardiovascular risk factors (LDL-C, fat mass), BMI, and hip circumference. A negative correlation was revealed among adiponectin with BMI and muscle mass, respectively. High-sensitivity CRP was associated with HbA1c, fat mass, and BMI. In parallel, participants’ level of physical activity was negatively associated with fat mass but not with leptin, adiponectin, hs-CRP, and IL-6. As such, we propose that leptin, adiponectin and hs-CRP may be used in this specific study population as prognostic indicators of cardiovascular risk.

## Figures and Tables

**Table 1:** Basic demographic and clinical parameters of study population.

Variables	*n* (%), Mean ± SD	Median (Range)
Gender (female)	36 (45%)/44 (55%)	
Chronological age (years)	14.89 ± 3.44	14.9 (12.1, 14.9)
Tanner stage (II–V)	11/9/8/52	
Height (cm)	160 ± 22.5	164 (153, 170.5)
Weight (Kg)	56.9 ± 15.5	58.3 (46.3, 69.1)
BMI (Kg/m^2^)	21.3 ± 3.6	21.3 (18.6, 23.2)
z score BMI (SD)	0.49 ± 0.93	0.43 (−0.072, 1.008)
Waist (cm)	74.3 ± 10.5	74 (66, 82)
Hip (cm)	50.75 ± 8.9	51 (45.5, 55)
Waist to hip ratio	1.48 ± 0.2	1.49 (1.36, 1.60)
Waist to height ratio	0.94 ± 4.3	0.45 (0.42, 0.49)
Fat mass percentage (%)	20.8 ± 7.4	20.7 (16.5, 25.6)
Muscle mass (kg)	45 ± 12.9	42.3 (36.3, 54.05)
Age at disease diagnosis (years)	9.02 ± 3.5	9.58 (7.1, 12)
Disease duration (years)	5.8 ± 4.03	5.54 (2.25, 8.29)
Insulin (iu/Kg/day)	0.84 ± 0.24	0.83 (0.68, 0.97)
Mean annual HbA1c (%)	8.0 ± 1.41	7.73 (7.05, 8.95)
Physical activity (7 days total steps count)	49,708 ± 24,033	45,171 (31,302, 64,053)

Abbreviations: SD: Standard Deviation; BMI: Body mass index; HbA1c: Hemoglobin A1c; T1D: Type-1-Diabetes.

**Table 2:** Biochemical and hematological parameters of study population.

*n* (%)	Mean ± SD	Median (Range)
Triglycerides (mg/dL)	69.9 ± 46.2	61 (45, 78)
Total cholesterol (mg/dL)	153.29 ± 27.67	153 (134, 169)
HDL-C (mg/dL)	60.4 ± 14	58 (49, 68)
LDL-C (mg/dL)	84.7 ± 23.2	83 (69, 101)
Lp(a) (mg/dL)	28.5 ± 44.5	13 (6, 24.6)
Leptin (ng/mL)	9.5 ± 10.6	5.4 (2.4, 12.6)
Squared leptin	2.66 ± 1.58	2.3 (1.5, 3.5)
Adiponectin (ng/mL)	32,295 ± 27,519	21,621 (15,290, 42,581)
Log adiponectin	10.13 ± 0.67	9.98 (9.63, 10.65)
Leptin/adiponectin	0.00045 ± 0.0006	0.0002 (0.00007, 0.0005)
Log leptin/adiponectin	−8.59 ± 1.51	−8.33 (−9.55, −7.53)
IL-6 (pg/mL)	16.16 ± 34.89	6.15 (5.04, 10.58)
1/IL-6	0.14 ± 0.07	0.16 (0.09, 0.19)
hs-CRP (mg/dL)	1524 ± 2541	816 (289, 1574)
Log hs-CRP	6.56 ± 1.22	6.70 (5.66, 7.36)
White blood count (/mm^3^)	6159 ± 1431	5980 (5300, 6900)
Neutrophils percentage (%)	47.73 ± 10.23	48 (41, 54.6)
Leukocytes percentage (%)	39.81 ± 9.47	40.5 (34, 47.6)
Platelets (/mm^3^)	249,052 ± 68,900	53,500 (214,000, 287,000)

Abbreviations: SD: Standard Deviation; HDL-C: High Density Lipoprotein-Cholesterol; LDL-C: Low Density Lipoprotein-Cholesterol; Lp(a): Lipoprotein (a); IL-6: Interleukin 6; hs-CRP: high-sensitivity C-Reactive Protein.

**Table 3:** Leptin correlations with basic demographic and physical and biochemical parameters with the use of univariant linear regression.

Px	Beta	(95% C.I. of Beta)	T-Statistic	*p*-Value
**Demographic and anthropometric parameters**				
Gender (female)	1.643	(1.035, 2.252)	5.38	<0.001
Chronological age (years)	0.033	(−0.069, 0.136)	0.64	0.522
Tanner stage (II–V)	0.2408	(−0.072, 0.554)	1.53	0.130
Height (cm)	0.002	(−0.013, 0.018)	0.32	0.751
Weight (kg)	0.02	(−0.002, 0.042)	1.78	0.078
Waist circumference (cm)	0.013	(−0.019, 0.047)	0.83	0.411
Hip circumference (cm)	0.044	(0.005, 0.083)	2.28	0.025
Waist to hip ratio	−2.017	(−3.734, −0.299)	−2.34	0.022
Waist to height ratio	−0.038	(−0.121, 0.044)	−0.92	0.359
ΒΜΙ (Kg/m^2^)	0.184	(0.096, 0.271)	4.21	<0.001
z score BMI (SD)	0.617	(0.262, 0.973)	3.46	0.001
Fat mass percentage (%)	14.07	(10.5001, 17.6526)	7.84	<0.001
Muscle mass (kg)	−0.008	(−0.036, 0.018)	−0.64	0.527
HbA1c (%)	0.139	(−0.098, 0.377)	1.17	0.247
Mean annual HbA1c (%)	0.0618	(−0.199, 0.323)	0.47	0.639
Number of daily glucose measurements	0.0267	(−0.071, 0.124)	0.54	0.589
Total number of weekly hypoglycemic episodes	0.009	(−0.043, 0.062)	0.35	0.726
Insulin (units/kg per day)	1.162	(−0.291, 2.616)	1.59	0.115
T1D duration (years)	0.02	(−0.06, 0.11)	0.64	0.527
Family situation * (family members living with the patient)	0.17	(−0.32, 0.68)	0.70	0.486
Insulin regime ** (mode of insulin administration)	0.719	(−0.005, 1.445)	1.98	0.052
Physical activity (7 days total steps count)	−6.29 × 10^−6^	(−0.00002, 8.49 × 10^−6^)	−0.85	0.400
**Laboratory parameters**				
White blood count (/mm^3^)	0.0002	(−0.00004, 0.0004)	1.65	0.103
Neutrophils percentage (%)	0.0373	(0.002, 0.072)	2.11	0.038
Leukocytes percentage (%)	−0.072	(−0.108, −0.036)	−4.03	<0.001
Platelets (/mm^3^)	4.25 × 10^−6^	(−9.90 × 10^−7^, 9.49 × 10^−6^)	1.62	0.110
Ferritin (ng/mL)	−0.025	(−0.05, −0.001)	−2.35	0.041
Urea (mg/dL)	−0.053	(−0.091, −0.01)	−2.84	0.006
Triglycerides (mg/dL)	0.0009	(−0.007, 0.009)	0.22	0.824
Total cholesterol (mg/dL)	0.009	(−0.002, 0.02)	1.54	0.128
HDL-C (mg/dL)	0.004	(−0.021, 0.03)	0.37	0.712
LDL-C (mg/dL)	0.021	(0.006, 0.036)	2.90	0.005
Lp(a) (mg/dL)	0.004	(−0.004, 0.013)	1.01	0.319

* Lives with both parents, only with mother or with father, with grandparents, with others or by himself/herself. ** Basal/bolus vs. pump regime. Abbreviations: (95% C.I.): 95% Confidence Interval; BMI: Body mass index; T1D: Type-1-Diabetes; HbA1c: Hemoglobin A1c, HDL-C: High Density Lipoprotein-Cholesterol; LDL-C: Low Density Lipoprotein-Cholesterol; Lp(a): Lipoprotein (a). Data are presented as the variable of interest, beta (95% C.I. of beta), T-statistic, and *p*-value.

**Table 4:** Adiponectin correlations with basic demographic and physical and biochemical parameters with the use of univariant linear regression.

Px	Beta	(95% C.I. of Beta)	T-Statistic	*p*-Value
**Demographic and anthropometric parameters**				
Gender (female)	0.179	(−0.012, 0.483)	1.18	0.242
Chronological age (years)	−0.058	(−0.1006, −0.015)	−2.73	0.008
Tanner (II–V)	−0.124	(−0.258, 0.009)	−1.86	0.067
Height (cm)	−0.01	(−0.016, −0.004)	−3.30	0.001
Weight (kg)	−0.013	(−0.022, −0.003)	−2.83	0.006
Waist circumference (cm)	−0.016	(−0.0303, −0.0019)	−2.27	0.026
Hip circumference (cm)	−0.013	(−0.0309, 0.003)	−1.62	0.109
Waist to hip ratio	0.022	(−0.747, 0.792)	0.06	0.954
Waist to height ratio	0.048	(0.013, 0.082)	2.80	0.006
ΒΜΙ (Kg/m^2^)	−0.056	(−0.096, −0.017)	−2.86	0.005
z score BMI (SD)	−0.109	(−0.271, 0.053)	−1.34	0.184
Fat mass percentage (%)	−0.703	(−2.7499, 1.34305)	−0.68	0.496
Muscle mass (kg)	−0.013	(−0.024, −0.002)	−2.38	0.020
HbA1c (%)	0.001	(−0.101, 0.10364)	0.03	0.980
Mean annual HbA1c (%)	−0.006	(−0.118, 0.104)	−0.12	0.904
Number of daily glucose measurements	0.012	(−0.029, 0.054)	0.59	0.558
Number of weekly hypoglycemic episodes	−0.003	(−0.026, 0.019)	−0.31	0.755
Insulin (units/kg per day)	−0.064	(−0.698, 0.569)	−0.20	0.840
T1D duration (years)	−0.036	(−0.073, 0.0008)	−1.95	0.055
Family situation * (family members living with the patient)	0.163	(−0.049, 0.377)	1.53	0.131
Insulin regime ** (mode of insulin administration)	0.113	(−0.205, 0.431)	0.71	0.481
Physical activity (7 days total steps count)	5.71 × 10^−7^	(−5.80 × 10^−6^, 6.94 × 10^−6^)	0.18	0.859
**Laboratory parameters**				
White blood count (/mm^3^)	−0.00004	(−0.0001, 0.00006)	−0.85	0.398
Neutrophils percentage (%)	−0.013	(−0.028, 0. 002)	−1.71	0.091
Leukocytes percentage (%)	0.004	(−0.012, 0.021)	0.50	0.621
Platelets (/mm^3^)	1.95 × 10^−6^	(−2.16 × 10^−7^, 4.11 × 10^−6^)	1.79	0.077
Ferritin (ng/mL)	−0.014	(−0.023, −0.005)	−3.58	0.005
Triglycerides (mg/dL)	−0.002	(−0.006, 0.0007)	−1.61	0.113
Total cholesterol (mg/dL)	0.0008	(−0.004, 0.006)	0.30	0.762
HDL-C (mg/dL)	0.002	(−0.009, 0.013)	0.41	0.683
LDL-C (mg/dL)	0.0005	(−0.006, 0.007)	0.14	0.886
Lp(a) (mg/dL)	−0.001	(−0.006, 0.002)	−0.84	0.407
Urea (mg/dL)	0.003	(−0.014, 0.02)	0.36	0.717

* Lives with both parents, only with mother or with father, with grandparents, with others or by himself/herself. ** Basal/bolus vs. pump regime. Abbreviations: (95% C.I.): 95% Confidence Interval; BMI: Body mass index; T1D: Type-1-Diabetes; HbA1c: Hemoglobin A1c; HDL-C: High Density Lipoprotein-Cholesterol; LDL-C: Low Density Lipoprotein-Cholesterol; Lp(a): Lipoprotein (a); hs-CRP: high-sensitivity C-Reactive Protein. Data are presented as the variable of interest, beta (95% C.I. of beta), T-statistic, and *p*-value.

**Table 5:** hs-CRP correlations with basic demographic and physical and biochemical parameters with the use of univariant linear regression.

Px	Beta	(95% C.I. of Beta)	T-Statistic	*p*-Value
**Demographic and anthropometric parameters**				
Gender (female)	0.234	(−0.31, 0.783)	0.85	0.399
Chronological age (years)	0.137	(0.063, 0.211)	3.70	<0.001
Tanner (II–V)	0.398	(0.169, 0.627)	3.46	0.001
Height (cm)	0.014	(0.002, 0.026)	2.39	0.019
Weight (kg)	0.035	(0.019, 0.051)	4.42	<0.001
Waist circumference (cm)	0.045	(0.021, 0.069)	3.76	<0.001
Hip circumference (cm)	0.061	(0.033, 0.089)	4.36	<0.001
Waist to hip ratio	−0.847	(−2.2195, 0.52508)	−1.23	0.223
Waist to height ratio	−0.055	(−0.119, 0.007)	−1.75	0.085
ΒΜΙ (Kg/m^2^)	0.186	(0.124, 0.247)	5.99	<0.001
z-score BMI (SD)	0.4694	(0.193, 0.745)	3.38	0.001
Fat mass percentage (%)	5.2859	(1.7809, 8.79103)	3.00	0.004
Muscle mass (kg)	0.027	(0.007, 0.048)	2.71	0.008
HbA1c (%)	0.228	(0.0504, 0.406)	2.55	0.013
Mean annual HbA1c (%)	0.258	(0.066, 0.449)	2.68	0.009
Number of daily glucose measurements	−0.09	(−0.172, −0.02)	−2.74	0.008
Number of weekly hypoglycemic episodes	−0.016	(−0.057, 0.024)	−0.82	0.415
Insulin (units/kg per day)	−0.158	(−1.301, 0.98)	−0.28	0.784
T1D duration	0.064	(−0.001, 0.131)	1.93	0.057
Family situation * (family members living with the patient)	0.131	(−0.258, 0.521)	0.67	0.503
Insulin regime ** (mode of insulin administration)	0.11	(−0.464, 0.685)	0.38	0.704
Physical activity (7 days total steps count)	3.98 × 10^−6^	(−7.48 × 10^−6^, 0.00001)	0.69	0.492
**Laboratory parameters**				
White blood count (/mm^3^)	0.0001	(−0.00005, 0.0003)	1.45	0.152
Neutrophils percentage (%)	0.0321	(0.006, 0.058)	2.45	0.017
Leukocytes percentage (%)	−0.0219	(−0.0502, 0.0064)	−1.54	0.128
Platelets (/mm^3^)	2.71 × 10^−6^	(−1.45 × 10^−6^, 6.88 × 10^−6^)	1.30	0.199
Ferritin (ng/mL)	0.0012	(−0.0179, 0.0204)	0.15	0.886
Urea (mg/dL)	−0.0141	(−0.045, 0.017)	−0.90	0.370
Triglycerides (mg/dL)	0.0016	(−0.004, 0.008)	0.52	0.607
Total Cholesterol (mg/dL)	0.003	(−0.007, 0.013)	0.59	0.558
HDL-C (mg/dL)	−0.009	(−0.029, 0.011)	−0.89	0.374
LDL-C (mg/dL)	0.011	(−0.001, 0.023)	1.83	0.072
Lp(a) (mg/dL)	−0.002	(−0.011, 0.005)	−0.68	0.501

* Lives with both parents, only with mother or with father, with grandparents, with others, or by himself/herself, ** Basal/bolus vs. pump regime. Abbreviations: (95% C.I.): 95% Confidence Interval; BMI: Body mass index; T1D: Type-1-Diabetes; HbA1c: Hemoglobin A1c; HDL-C: High Density Lipoprotein-Cholesterol; LDL-C: Low Density Lipoprotein-Cholesterol; Lp(a): Lipoprotein (a); hs-CRP: high-sensitivity C-Reactive Protein. Data are presented as the variable of interest, beta (95% C.I. of beta), T-statistic, and *p*-value.

**Table 6:** Multiple regression models of muscle mass, after applying the forward stepwise method for variable insertion and evaluation with demographic and anthropometric data.

Multiple Model Factor of Interest	Overall Model F, R^2^, DF, p	Factor Beta (95% C.I.)	Factor SE	Factor T-Statistic	Factor *p*-Value
**Muscle mass**					
**Regression equation** **Muscle mass = −0.69 + 2.21 × z score BMI −8.24 × gender + 1.47 × age + 3.67 × Tanner + 0.26 × waist circumference**					
z score BMI (SD)	114.28, 0.8867, 78, <0.001	2.21 (0.69, 3.74)	0.764	2.90	0.005
Gender (female)		−8.24 (−10.54, −5.94)	1.152	−7.15	<0.001
Age (years)		1.47 (0.912, 2.033)	0.281	5.24	<0.001
Tanner (II–V)		3.67 (2.07, 5.28)	0.805	4.56	<0.001
Waist (cm)		0.26 (0.098, 0.429)	0.082	3.18	0.002

Abbreviations: F: overall model F-statistic; R^2^: overall model R squared statistic; DF: Degrees of Freedom; beta: factor beta regression coefficient; (95% C.I.): 95% Confidence Interval; SE: Standard Error; BMI: Body mass index.

**Table 7:** Multiple regression models of fat mass percentage, after applying the forward stepwise method for variable insertion and evaluation with demographic and anthropometric data.

Multiple Model Factor of Interest	Overall Model F, R^2^, DF, p	Factor Beta (95% C.I.)	Factor SE	Factor T-Statistic	Factor *p*-Value
**Fat mass percentage (%)**					
**Regression equation** **Fat mass percentage = 0.072 + 0.08 × gender + 0.03 × z-score BMI −3.9 × 10^7^ × total steps −0.15 × 1/IL6 + 0.007 × squared leptin**					
Gender (female)	51.26, 0.7760, 79, <0.001	0.0895 (0.06909, 0.10994)	0.01025	8.73	<0.001
z score BMI (SD)		0.03997 (0.2993, 0.05001)	0.0050387	7.93	<0.001
Physical activity (7 days total steps count)		−3.90 × 10^−7^ (−7.33 × 10^−7^, −4.66 × 10^−8^)	1.72 × 10^−7^	−2.26	0.027
Inverse IL-6		−0.1572 (2,745,324, −0.0399454)	0.05886	−2.67	0.009
Squared leptin		0.0077 (0.00076, 0.01464)	0.00348	2.21	0.030

Abbreviations: F: overall model F-statistic; R^2^: overall model R squared statistic; DF: Degrees of Freedom; beta: factor beta regression coefficient; (95% C.I.): 95% Confidence Interval; SE: Standard Error; BMI: Body mass index; IL-6: Interleukin 6.

**Table 8:** Multiple regression models of leptin, adiponectin, and CRP, after applying the forward stepwise method for variable insertion and evaluation with demographic and anthropometric data.

Multiple Model Factor of Interest	Overall Model F, R^2^, DF, p	Factor Beta (95% C.I.)	Factor SE	Factor T-Statistic	Factor *p* Value
**Leptin (squared leptin)**					
**Regression equation** **Squared leptin = −5.0 + 0.04 × age + 1.50 × gender −0.04 × weight + 0.35 × BMI**					
Age (years)	19.66, 0.51, 79, <0.001	0.043 (−0.08, 0.16)	0.06	0.7	0.487
Gender (female)		1.50 (0.96, 2.04)	0.27	5.55	<0.001
Weight (kg)		−0.049 (−0.098, −0.0001)	0.024	−2.0	0.049
BMI (kg/m^2^)		0.351 (0.195, 0.506)	0.078	4.5	<0.001
**Adiponectin (log Adiponectin)**					
**Regression equation** **Log adiponectin = 10.88−0.053 × age + 0.043 × waist to height ratio**					
Age (years)	7.39, 0.16, 78, 0.0012	−0.53 (−0.095, −0.011)	0.021	−2.53	0.014
waist/height		0.043 (0.010, 0.077)	0.016	2.62	0.011
**hs-CRP (log hs-CRP)**					
**Regression equation** **Log hs-CRP = 3.99 + 0.15 × age + 0.53 × z score BMI**					
Age (years)	17.67, 0.31, 79, <0.001	0.154 (0.087, 0.222)	0.033	4.58	<0.001
z score BMI (SD)		0.537 (0.288, 0.785)	0.124	4.31	<0.001

Abbreviations: F: overall model F-statistic; R^2^: overall model R squared statistic; DF: Degrees of Freedom; beta: factor beta regression coefficient; (95% C.I.): 95% Confidence Interval; SE: Standard Error; BMI: Body mass index; hs-CRP: high-sensitivity C-Reactive Protein.

## Data Availability

The authors confirm that the majority of the data supporting the findings of this study are available within the article. Raw data are available from the authors upon reasonable requests.
